# A Role for Insulin in Diabetic Neuropathy

**DOI:** 10.3389/fnins.2016.00581

**Published:** 2016-12-23

**Authors:** Caleb W. Grote, Douglas E. Wright

**Affiliations:** Department of Anatomy and Cell Biology, University of Kansas Medical CenterKansas City, KS, USA

**Keywords:** insulin, insulin resistance, neuropathy, pain, neurotrophic factors, diabetes complications

## Abstract

The peripheral nervous system is one of several organ systems that are profoundly affected in diabetes. The longstanding view is that insulin does not have a major role in modulating neuronal function in both central and peripheral nervous systems is now being challenged. In the setting of insulin deficiency or excess insulin, it is logical to propose that insulin dysregulation can contribute to neuropathic changes in sensory neurons. This is particularly important as sensory nerve damage associated with prediabetes, type 1 and type 2 diabetes is so prevalent. Here, we discuss the current experimental literature related to insulin's role as a potential neurotrophic factor in peripheral nerve function, as well as the possibility that insulin deficiency plays a role in diabetic neuropathy. In addition, we discuss how sensory neurons in the peripheral nervous system respond to insulin similar to other insulin-sensitive tissues. Moreover, studies now suggest that sensory neurons can also become insulin resistant like other tissues. Collectively, emerging studies are revealing that insulin signaling pathways are active contributors to sensory nerve modulation, and this review highlights this novel activity and should provide new insight into insulin's role in both peripheral and central nervous system diseases.

## Introduction

Approximately 25.8 million people in the United States are diagnosed with diabetes, and this number is expected to almost double by 2030 (Wild et al., 2004; CDC, National Diabetes Fact Sheet, 2014). With this dramatic increase in diabetes also comes a dramatic increase in the complications associated with diabetes. Damage to the peripheral nervous system due to diabetes is associated with a particularly high level of morbidity and afflicts over 50% of all diabetic patients (Zochodne, 2007; CDC, National Diabetes Fact Sheet, 2014). Diabetic neuronal complications first present in the distal extremities and can result in either numbness or chronic pain; and is one of the major factors in the development of Charcot joints, foot ulcers, and limb amputation in diabetic patients (Zochodne, 2007). The current treatment for DN involves only symptomatic relief, and often the results are disappointing (Apfel et al., 2000; Chalk et al., 2007; Habib and Brannagan, 2010). Defining the pathogenic mechanisms that contribute to diabetic neuropathy (DN) is essential to establish both appropriate pharmacological and nonpharmacological treatments for this devastating diabetic complication. The currently investigated pathways of DN pathogenesis mainly focus on the cellular damage associated with the various cascades activated in response to hyperglycemia (for review see Tomlinson and Gardiner, 2008; Vincent et al., 2011), including reactive oxygen species (ROS), advanced glycation end-products (AGE), and polyol flux. However, there are 2 major insults in diabetes. The first is the loss of insulin signaling, either due to insulinopenia (type 1), or insulin resistance (type 2), and secondly, is the resultant elevated blood glucose levels. Accordingly, the role of direct insulin singling on sensory neurons and how disruption of this signaling may be a contributing factor to DN pathogenesis has been the subject of several studies which are reviewed here.

## Insulin receptor signaling

**Figure 1:**
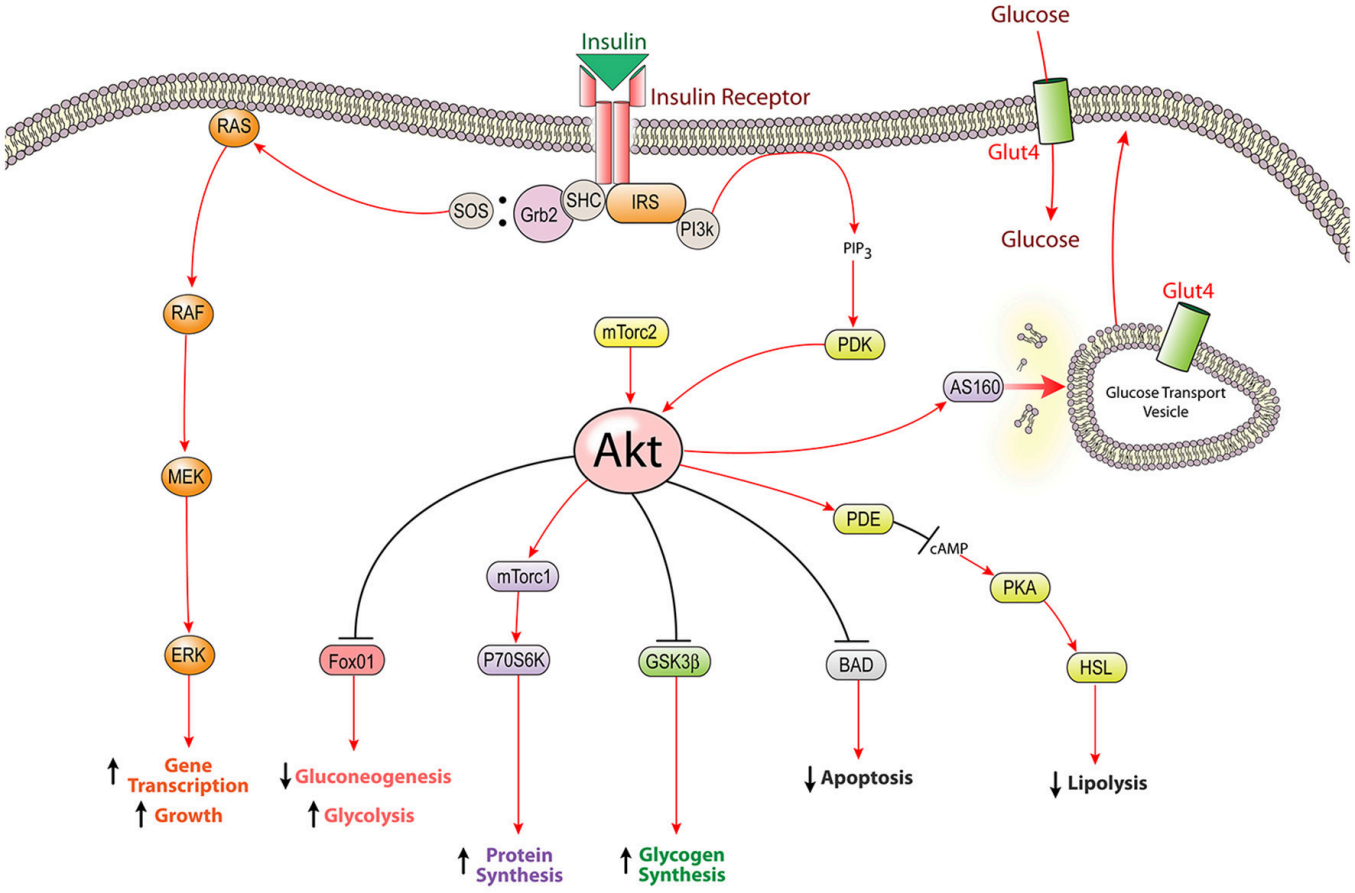
**The insulin signaling pathway: intracellular insulin signaling is initiated by insulin receptor tyrosine kinase activity leading to the activation of both the PI3K-Akt pathway and the MAPK pathway**. The most well characterized function of insulin signaling is glucose homeostasis; however insulin stimulates several other cellular mechanisms including the biosynthesis of proteins, glycogen, and lipids. Insulin receptor substrate (IRS), glycogen synthetase kinase β (GSK3β), extracellular signal-related kinase (ERK), Srchomology-2-containing protein (Shc), forkhead box protein O1 (FOXO1), Akt substrate of 160 kDa (AS160), mammalian target of rapamycin complex (mTorc), p70 ribosomal protein S6 kinase (p70S6K), hormone sensitive lipase (HSL), phosphodiesterase (PDE), protein kinase A (PKA), phosphoinositide dependent kinase (PDK), Growth factor receptor-bound protein 2 (Grb2), son of sevenless (SOS).

The insulin signaling cascade is propagated by phosphorylation events beginning with activation of the insulin receptor tyrosine kinase upon insulin binding. After activation, the insulin receptor kinase phosphorylates tyrosine residues on both the receptor and docking proteins, such as insulin receptor substrate (IRS). Tyrosine phosphorylation allows downstream mediators with src homology-2 (Sh2) domains to bind IRS and localize to the plasma membrane. Two key Sh2 containing mediators are PI3-kinase, which activates the Akt cascade, and Grb2/SOS, which activates the MAPK cascade (White, 2002, 2006). These effectors eventually lead to increased transcription, translation, and translocation of the proteins necessary to carry out insulin's actions (Figure 1).

Insulin has numerous effects throughout the body, mostly relating to energy storage and glucose homeostasis. In peripheral “insulin-sensitive” tissues (liver, muscle, and adipose), insulin mediates glucose metabolism by stimulating glucose uptake through translocation of glut4, as well as controlling glucose breakdown and synthesis via its effects on glycolysis and gluconeogenesis. Additionally, insulin promotes glycogen synthesis through inhibition of glycogen synthetase kinase, increases protein production through mTor activation, as well as promotes fatty acid synthesis and inhibits lipolysis through activation of Acetyl-CoA Carboxylase and inhibition of hormone sensitive lipase, respectively. Furthermore, insulin can modulate gene transcription through the MAPK pathway or through Akt-mediated phosphorylation of FOXO transcription factors which results in nuclear exclusion and a reduction in FOXO-mediated gene expression (Le Roith and Zick, 2001; Taniguchi et al., 2006). Beyond energy balance, insulin plays a role in several other aspects of physiology, including: fertility, blood lipid levels, blood pressure, as well as growth and survival of pancreatic beta cells, bone, retina, and neurons (Skaper et al., 1982; Recio-Pinto et al., 1986; White, 2002; Takamoto et al., 2008).

## Insulin and the nervous system

Throughout history, insulin signaling in the nervous system has been fairly ignored, because unlike muscle and adipose tissues, the majority of neurons do not take up glucose in an insulin dependent manner (Greene and Winegrad, 1979; Patel et al., 1994). However, neurons do express insulin receptors (Plum et al., 2005). Neurons primarily express the glut1 and glut3 glucose transporters and take up glucose via a concentration gradient receptor mediated process (Choeiri et al., 2002). However, it has recently been demonstrated that certain areas of the brain, such as the olfactory bulb, hippocampus, and hypothalamus express glut4 transports (Leloup et al., 1996). Glucose is the main energy supply for neurons; however, in states of extreme starvation neurons can utilize ketone bodies (Sokoloff, 1973).

Insulin crosses the blood-brain barrier (BBB) through a saturable receptor mediated transport system, termed receptor-mediated transcytosis (Baura et al., 1993). During this process, serum insulin binds the insulin receptor on the endothelial cells of the blood brain barrier; the ligand-receptor complex is internalized and transported to the opposite side of the cell where insulin is released. Insulin receptor-mediated transcytosis is currently one of the most targeted systems in drug development to transport chemicals across the BBB (Wang et al., 2009).

Diabetes has recently been implicated as a risk factor for several neurological diseases, including Alzheimer's and Parkinson's disease (Luchsinger et al., 2001; Hu et al., 2007; Jolivalt et al., 2008, 2010, 2012; Fadel et al., 2013). This has led to increased interest in the role that insulin might play in the nervous system and it is becoming very clear that there is a strong link between diabetes and Alzheimer's disease (Ribe and Lovestone, 2016). Many of the CNS changes associated with diabetes are similar to those observed in Alzheimer's disease, including increased beta amyloid and tau phosphorylation (Akter et al., 2011). Additionally, the brains from Alzheimer's patients show characteristic signs of insulin resistance (Talbot et al., 2012) and the insulin sensitizing drugs, thiazolidinediones, have been shown to improve memory in both mice (Pedersen et al., 2006) and human patients (Sato et al., 2011). Furthermore, both intracerebroventricular and intrahippocampal insulin administration has been demonstrated to improve memory formation in rats (Park et al., 2000; Haj-ali et al., 2009; McNay et al., 2010), and insulin has been demonstrated to regulate synapse number and plasticity (Chiu et al., 2008). Recently, in phase 1 clinical trials, Alzheimer's patients that received intranasal insulin treatment demonstrated improved memory and activities of daily living (Craft et al., 2012).

Experimental studies that have addressed the role of insulin signaling in the CNS reveal that removal of insulin signaling in the hippocampus using viral delivery of insulin receptor antisense sequences impaired hippocampal plasticity related to long-term potentiation and spatial learning of rats (Grillo et al., 2015). Neuronal knockout of the insulin receptor in the CNS using Cre-recombinase driven by a nestin promoter led to a loss in kinase activation and inhibition of apoptosis. Overall, this reduced phosphorylation of AKt and GSK3β, which impacted Tau phosphorylation, an important component of Alzheimer's disease (Schubert et al., 2004). In type 1 diabetic rats, the hippocampus was shown to be more responsive to insulin administration compared to nondiabetic rats, and sustained insulin delivery to diabetic rats could normalize insulin receptor activity, suggesting that insulin treatment as an intervention may provide benefits to cognitive and memory deficits associated with CNS disease (King et al., 2015).

Beyond a role in memory and cognition, insulin is involved in centrally regulating glucose metabolism and food intake via signaling in the hypothalamus. Insulin inhibits neuronal firing of the NP-Y/AgRP neurons of the arcuate nucleus by activating K_ATP_ leading to neuronal hyperpolarization, resulting in decreased release of the orexigenic hormone NP-Y (Spanswick et al., 2000; Gerozissis, 2008). Additionally, this process has been demonstrated to centrally regulate liver gluconeogenesis, and is hypothesized to be a major mechanism contributing to obesity and insulin resistance (Obici et al., 2002a,b). There also appears to be a role for insulin signaling in the spinal cord. Insulin receptors are expressed in dorsal and ventral spinal cord neurons (Sugimoto et al., 2002) and insulin has also been demonstrated to regulate AMPA-induced neuronal damage (Kim and Han, 2005) and modulate AMPA excitatory currents in the spinal cord dorsal horn (Spicarova and Palecek, 2010). It is known that insulin receptors are expressed Additionally, insulin receptor signaling in the CNS has been shown be involved in regulating neuronal development (Chiu and Cline, 2010). Overall, these observations suggest that although neurons do not take up glucose in an insulin dependent manner, many neuronal populations do seem to be insulin responsive and insulin may be important to maintaining proper neuronal function.

## Insulin as a neurotrophic factor in the PNS

Insulin is a member of the insulin-like super family that includes Insulin, IGF1, and IGF2. While IGF1 has been a well-defined neurotrophic factor for some time, insulin's effect on neurons has only gained significant attention over the past 15–20 years. A growing body of literature has now established insulin as a potent neurotrophic factor that appears essential to promoting proper neuronal function. Insulin receptors are expressed on both the DRG neuron soma as well as in the peripheral nerve (Sugimoto et al., 2000, 2002; Shettar and Muttagi, 2012). Several reports have indicated that the insulin receptor is predominantly expressed in small nociceptive neurons. Baiou et. al. indicated that approximately 40% of DRG neurons express the insulin receptor and that approximately 75% of insulin receptor expressing neurons were co-labeled with peripherin (Baiou et al., 2007). TRPV1 co-labeling indicated 25% of all DRG neurons coexpress TRPV1 and the insulin receptor and that approximately 68% of TRPV1 positive neurons express the insulin receptor (Baiou et al., 2007). Furthermore, insulin receptor expression was not confined to one c-fiber subtype, as insulin receptor expressing neurons were co-labeled with either CGRP (peptidergic) or IB4 (non-peptidergic). However, and in contrast, a number of reports have also reported strong co-labeling with neurofilament-H, a marker of large myelinated neurons (Guo et al., 2011; Singh et al., 2012).

In primary culture models of embryonic sympathetic and sensory neurons, insulin supplementation has been shown to have many functional effects (Snyder and Kim, 1980; Bothwell, 1982; Recio-Pinto et al., 1986). Insulin stimulation appears to increase neuritogenesis, as well as neurite length and area. Recio-Pinto et al. showed that the percent of both sympathetic and sensory neurons bearing neurites increased in a dose dependent manner with insulin supplementation, with an ED_50_ of 0.4 nM for embryonic chick sympathetic neurons and an ED_50_ of 30 nM for dorsal root ganglion (DRG) sensory neurons (Recio-Pinto et al., 1986). This study provides evidence that insulin may act as a developmental neurotrophic factor, however, detailed studies of the developmental actions of insulin need further study, particularly in light that insulin does not appear to be as potent as other traditional neurotrophic factors like nerve growth factor (Recio-Pinto et al., 1986). In adult DRG neurons, Fernyhough et al. reported that insulin increased the rate of neurite regeneration in rat DRG cultures 3.5-fold compared to control cultures without insulin supplementation (Fernyhough et al., 1993). Interestingly, this effect appears to be additive with NGF supplementation (Recio-Pinto et al., 1984; Jones et al., 2003). One possible mechanism through which insulin may be promoting an increase in neurite outgrowth is through stabilization of tubulin microtubule mRNA, an essential part of neurite formation, as suggested by Fernyhough et al. (1989). Beyond neurite outgrowth, many reports also noted an apparent increase in neuronal survival with insulin supplementation in cultured adult sympathetic neurons, sensory neurons, and SH-SY5Y cells (Recio-Pinto et al., 1986; Li et al., 2003), and insulin is characterized to be one of the few essential molecules required for cultured primary peripheral neurons (Skaper et al., 1982). Furthermore, stimulation of the PNS with insulin has shown strong activation of the PI3K-Akt pathway, a pathway that is directly related to axonal growth and neuronal survival (Huang et al., 2005). Thus, a possible molecular mechanism of increased neuronal survival with insulin supplementation may be through insulin-induced Akt activation, which in turn shuts down apoptosis through inhibition of both BAD and caspase 9 (Datta et al., 1997).

Additionally, recent evidence has demonstrated that insulin may play an important role in Schwann cell physiology and Schwann cell dysfunction has been implicated in diabetic neuropathy (Eckersley, 2002; Song et al., 2003). Schwann cells express the insulin receptor in the basal lamina, plasma membrane and cytoplasmic processes (Shetter et al., 2011) and respond to insulin treatment (King et al., 2015). Insulin receptor expression in Schwann cells during development parallels myelin glycoprotein P zero (P0) expression and growth of the myelin sheath. Furthermore, insulin supplementation can induce P0 expression in primary Schwann cell culture, indicating that insulin could have crucial roles in myelination and peripheral nerve support via Schwann cell signaling (Shettar and Muttagi, 2012). Moreover, Schwann cells Akt activation can induce proliferation (Fex Svenningsen and Kanje, 1996), differentiation (Ogata et al., 2004) and myelination (Liang et al., 2007). Finally, insulin can modify myelin protein expression in the setting of diabetic neuropathy (Rachana et al., 2016).

Interestingly, it has been demonstrated that insulin may modify TRPV1 sensitivity and membrane expression (Van Buren et al., 2005; Lilja et al., 2007) and alterations in TRPV1 have been reported in DN (Hong and Wiley, 2005; Pabbidi et al., 2008). Several studies have demonstrated that insulin can sensitize and potentiate TRPV1 signaling by lowering the threshold for activation and increasing membrane translocation (Sathianathan et al., 2003; Van Buren et al., 2005; Lilja et al., 2007).

Beyond its effects on neurons *in vitro*, insulin has also been shown to have dramatic neurotrophic qualities *in vivo* and *in vivo* insulin signaling was recently demonstrated throughout the PNS in response to IP injection (Grote et al., 2013a). In nerve injury models (nerve transection or nerve crush), recovery from the ensuing pathological changes is accelerated by insulin supplementation. Xu et al. showed that intraperitoneal (IP) injections of insulin (0.02 IU Humulin R) twice daily increased both the rate of motor endplate reinnervation (measured by M wave amplitude) and hindpaw motor function recovery after sciatic nerve transection (Xu et al., 2004). Furthermore, in these studies it was also demonstrated that systemic insulin treatment through IP injections increased the number of mature regenerating myelinated fibers after nerve crush. Mice in the insulin treated group displayed significantly increased axonal and fiber diameter as well as increased axonal area (Xu et al., 2004). Similar results were reported in a comprehensive study comparing the effects of intrathecal (through mini-osmotic pump) or near nerve insulin treatment on peripheral nerve regeneration after nerve crush injury by Toth et al. (2006a). In the experimental paradigm of this study, the most dramatic effects of insulin treatment were observed in the group receiving intrathecal insulin. In separate experiments of sural (mostly sensory axons) and peroneal (mostly motor axons) nerve crush, insulin supplementation prevented degeneration of axons proximal to the nerve injury and accelerated regeneration of axons distal to the crush site. These changes were associated with an increase in axonal fiber density, size, and regenerating fiber clusters in both the sural and peroneal nerve with intrathecal insulin treatment. Furthermore, these observed morphological differences were coupled with an increase in CGRP and translated into an accelerated recovery of thermal sensation after nerve injury in insulin treated rats. Additional studies of IGF have also demonstrated a role for impaired IGF signaling in motor neurons in diabetic neuropathy (Simon et al., 2015; Rauskolb et al., 2017). Viral delivery of IGF1 to models of diabetic neuropathy also revealed positive actions of IGF1 perhaps through vascular endothelial growth factor expression (VEGF), and signaling via Akt/PI3K pathways in sensory and motor nerves (Homs et al., 2014).

Collectively, these studies have established insulin and IGF1 as key components of neuronal support and have led to the assumption that disruptions in insulin availability (reduced circulating levels or reduced signaling) could have detrimental effects on neuronal function.

## Insulin and diabetic neuropathy

As previously discussed, the currently investigated pathways of DN pathogenesis mainly focus on the cellular damage associated with the various cascades activated in response to hyperglycemia, yet there is growing interest in the role of neuronal insulin signaling in DN development and progression (Zochodne, 2014, 2015). Epidemiologic data from the Diabetes Control and Complications Trial (DCCT) provided very strong evidence of the link between poor glucose control and DN (Diabetes Control Complications Trial (DCCT) Research Group, 1995a). Results from this trial indicated that patients with intensive glycemic control (3 or 4 daily insulin injections or external pump) showed a 64% percent reduction in neuropathy over a 5-year period as compared to patients on conventional therapy (1 or 2 daily insulin injections with mixed rapid and intermediating acting insulin). Furthermore, patients on conventional therapy experienced a steady deterioration in nerve conduction velocity, while patients in the intensive treatment group displayed no change and even a slight improvement (Diabetes Control Complications Trial (DCCT) Research Group, 1995a,b). Accordingly, the best known treatment for prevention of neuropathic complications is strict glycemic control. Interpretation of this clinical data has led to a large emphasis on the role of hyperglycemia in DN, however another interpretation of this data reveals that strict glycemic control also means a more balanced, steady, and physiological insulin exposure. Thus, the reduction in DN in intensive treatment group may be a result of restoration of the lost neuronal insulin signaling key to maintaining proper sensory function rather than just the control of hyperglycemia.

## Low insulin without hyperglycemia causes signs of DN

Due to the intimate connection between insulin and blood glucose levels; teasing out the consequences of changes in one variable without disruptions in the other is difficult. However, several studies have demonstrated that in instances of low serum insulin, yet euglycemia, abnormalities in sensory function develop. Correspondingly, strong evidence has also shown that low dose insulin treatment of animals with DN can reverse many of the abnormal morphologic and behavioral changes associated with the disease, without significantly altering glucose levels.

A common animal model of type 1 diabetes is to use the beta-cell toxin STZ to induce severe insulinopenia and thus hyperglycemia. However, there is a variable response to STZ and not all animals will develop hyperglycemia and diabetes. In 2010, Romanovsky et al. characterized a cohort of these euglycemic-STZ injected rats, and showed that while they did not have elevated glucose levels, they did have a significant decrease in serum insulin concentrations as compared to rats that received vehicle. Interestingly, euglycemic-STZ rats did display mechanical hyperalgesia indicated by a reduced threshold on a paw-pressure withdrawal test. These changes were similar to that of hyperglycemic-STZ rats, although hyperglycemic rats did maintain a lower threshold (Romanovsky et al., 2010). In an earlier study, it was also demonstrated that this observed change in paw-pressure threshold correlated significantly with insulin deficiency in euglycemic-STZ rats and could be ameliorated with low-dose insulin treatment (Romanovsky et al., 2006). The euglycemic-STZ rats showed no alterations in mechanical sensitivity in response to von Frey filaments, no change in thermal sensation, or decreases in nerve conduction velocity, perhaps indicating that the loss of neuronal insulin support and hyperglycemia contribute to different features of DN in the limbs. Recently, additional biomarkers of neuropathy outside of peripheral nerves in the limbs have been improved by insulin. Topical application of insulin to the cornea prevented the nerve depletion in the cornea (Chen et al., 2013).

Similar results of neuropathy without overt hyperglycemia have also been demonstrated in the Goto-Kakizaki (GK) rat. Murakawa et al. evaluated the effect of continued impaired glucose tolerance (IGT) and progressive insulinopenia, without severe hyperglycemia on peripheral neurophysiology and neuromorphology in the GK rat (Murakawa et al., 2002). While no differences in PNS function were observed in the 2-month-old GK rat with IGT and hyperinsulinemia, 18-month-old GK rats with IGT and insulinopenia displayed classical features of diabetic neuropathy (reduced NCV, loss of unmyelinated axons, and increased frequency of regenerating fibers). These neuropathic changes developed without overt fasting hyperglycemia in 18 month old GK rats (control = 3.2 ± 0.4 mM and GK = 4.4 ± 1.3 mM) and the authors suggest that these changes appear to be more related to the decrease in neuronal insulin support. Furthermore, in conjunction with the increase in CGRP expression with insulin treatment observed by Toth et al., Murakawa et al. noted a significant decrease in CGRP expression in insulinopenic 18-month-old GK rats (Murakawa et al., 2002; Toth et al., 2006b). Together these results suggest that one of the mechanisms through which insulin may promote proper sensory function is by maintaining synthesis of key neuromodulator proteins and peptides.

It has also been demonstrated that sequestering of endogenous intrathecal insulin in nondiabetic rats by intrathecally infusing anti-insulin antibodies produces slowed motor nerve conduction and atrophy of axonal fibers, similar to that seen in models of diabetic neuropathy (Brussee et al., 2004), again suggesting that non-glycemic triggers of DN exist and that the loss of PNS insulin signaling may be one of the initiating events. Finally, it has been reported that STZ-diabetic rats show reduced insulin receptor activation in the sciatic nerve (Sugimoto et al., 2008). The rapid change in insulin receptor signaling was correlative with the rapid onset of mechanical hyperalgesia. This was one of the first publications investigating insulin signaling in the sciatic nerve and the authors speculate that the change in sciatic nerve insulin signaling may help explain the change in nociceptive behavior associated with DN.

## Low dose insulin reverses signs of DN

Many studies have shown that a loss of PNS insulin signaling may contribute to DN, similarly, several reports have demonstrated that low-dose insulin (insufficient to reduce hyperglycemia) can have beneficial effects on the signs and symptoms of DN. Brussee et. al. demonstrated that intrathecal delivery of insulin or equimolar IGF1 daily for 4 weeks could not only restore both motor and sensory nerve conduction deficits, but also prevent axonal atrophy in type 1 diabetic rats (Brussee et al., 2004). In a similar experiment, both intrathecal insulin and IGF1 were able to reverse the loss of epidermal nerve fiber density and length in diabetic rats (Toth et al., 2006a), which is a well-documented and quantifiable consequence of the “dying back” neuropathy associated with diabetes. Subcutaneous insulin delivery within these same experimental paradigms did not alter the investigated neuronal parameters. Studies in diabetic rats also reported positive actions of subcutaneous low dose insulin on reducing peripheral nerve dysfunction and MAP kinase activity (Sugimoto et al., 2013). This is in contrast to the results from Hoybergs and Meert, which demonstrated that low-dose insulin delivered through subcutaneous insulin pellet can nearly normalize diabetes-induced tactile allodynia and mechanical hyperalgesia, despite persistent hyperglycemia (blood glucose levels dropped from 600 mg/dl to approximately 400 mg/dl 2 weeks after insulin pellet insertion; Hoybergs and Meert, 2007). Thus, some controversy still exists as the appropriate dosing regimen and delivery method most appropriate for beneficial effects on the PNS, however it does appear that insulin treatment can relieve symptoms of DN through mechanisms other than reducing elevated blood glucose levels.

Most recently, Guo et. al reported that intraplantar delivery of insulin at sub-glucose lowering levels not only reversed the loss of intraepidermal nerve fiber density but also slightly ameliorated some of the symptoms of DN (Guo et al., 2011). This study demonstrated the efficacy of local insulin administration on epidermal innervation in several mouse models of diabetic neuropathy, including type 1 diabetes induced by STZ in C57BL/6J, CD-1, and CFW as well as *db/db* type 2 diabetic mice. Intraplantar insulin showed a benefit on epidermal axons over vehicle control in each of these DN models and in diabetic C57BL/6J mice the increase in epidermal innervation with insulin treatment was also associated with upregulation of GAP43/B50, a growth associated protein. Along with changes in innervation, local insulin administration improved deficits in mechanical but not thermal sensation. These results further corroborate the neuronal growth promoting qualities of insulin and the potent affects that insulin treatment *in vivo* has on symptoms of DN.

Beyond its effects on sensorimotor behavior and epidermal innervation, some subcellular pathological changes associated with DN can be alleviated with insulin treatment. Defects in sensory neuron mitochondrial function has been investigated as a possible mechanism contributing to DN through several different pathways, including the over-production of ROS and reduced respiration through defects in the electron transport chain. Insulin and mitochondria are intimately connected through numerous metabolic pathways, and proper insulin signaling is essential for proper mitochondrial function (Cheng et al., 2010). However, attempts to demonstrate excessive mitochondrial ROS production in DRG neurons (Akude et al., 2011), Schwann cells (Zhang et al., 2010), and kidney (Dugan et al., 2013) have only revealed decreases in ROS or no change in the setting of diabetes. Insulin treatment has been shown to improve many of the mitochondrial defects associated with DN (Huang et al., 2003, 2005; Chowdhury et al., 2010). Huang et al. reported that in a STZ model of type 1 diabetes, DRG neuronal mitochondria display increased depolarization and Chowdhury et. al. reported that diabetes can induce deficits in mitochondrial respiration as well as mitochondrial protein expression (Huang et al., 2003; Chowdhury et al., 2010). In both of these reports, insulin supplementation restored the mitochondrial parameters back to the levels observed in control animals. It is clear that this topic requires further investigation to sort out whether reduced insulin signaling may be one of the compounding factors affecting proper mitochondrial function.

Similar to the beneficial roles of insulin treatment on sensory deficits associated with DN, insulin treatment has been shown to protect against late-stage diabetes-induced motor neuropathy as well (Francis et al., 2011). Intranasal insulin (and subcutaneous insulin to a lesser extent) showed beneficial effects on motoneuron morphology and function. Insulin treated diabetic mice (8 month old CD1) showed protection against electrophysiological decline, loss of neuromuscular junctions, and loss of motor function (as measured with forelimb and hindlimb grip testing as well as rearing activity). These results provided further evidence of the neurotrophic qualities of insulin and the potential impact it may have on proper neuronal function.

A link between reduced insulin signaling in type 1 (insulinopenic) and type 2 (hyperinsulinemic) diabetic models was established with the recent demonstration of PNS insulin resistance. *In vitro* studies using models of chronic insulin treatment and type 2 diabetic ob/ob and db/db mice have demonstrated blunted Akt activation in response to insulin as compared to controls in sensory neurons and these results were correlated with reduced neurite outgrowth (Grote et al., 2011) and changes in mitochondrial-associated proteins (Kim et al., 2011). Neurite outgrowth stimulated by insulin also appears to be sensitive to higher doses of insulin suggestive of insulin resistance (Singh et al., 2012). This idea of neuronal insulin resistance is in agreement with studies reporting reduced downstream insulin signaling *in vivo* in the PNS of insulin resistant ob/ob mice in response to either IT and IP injections of insulin (Grote et al., 2013b).

## Conclusions

**Figure 2:**
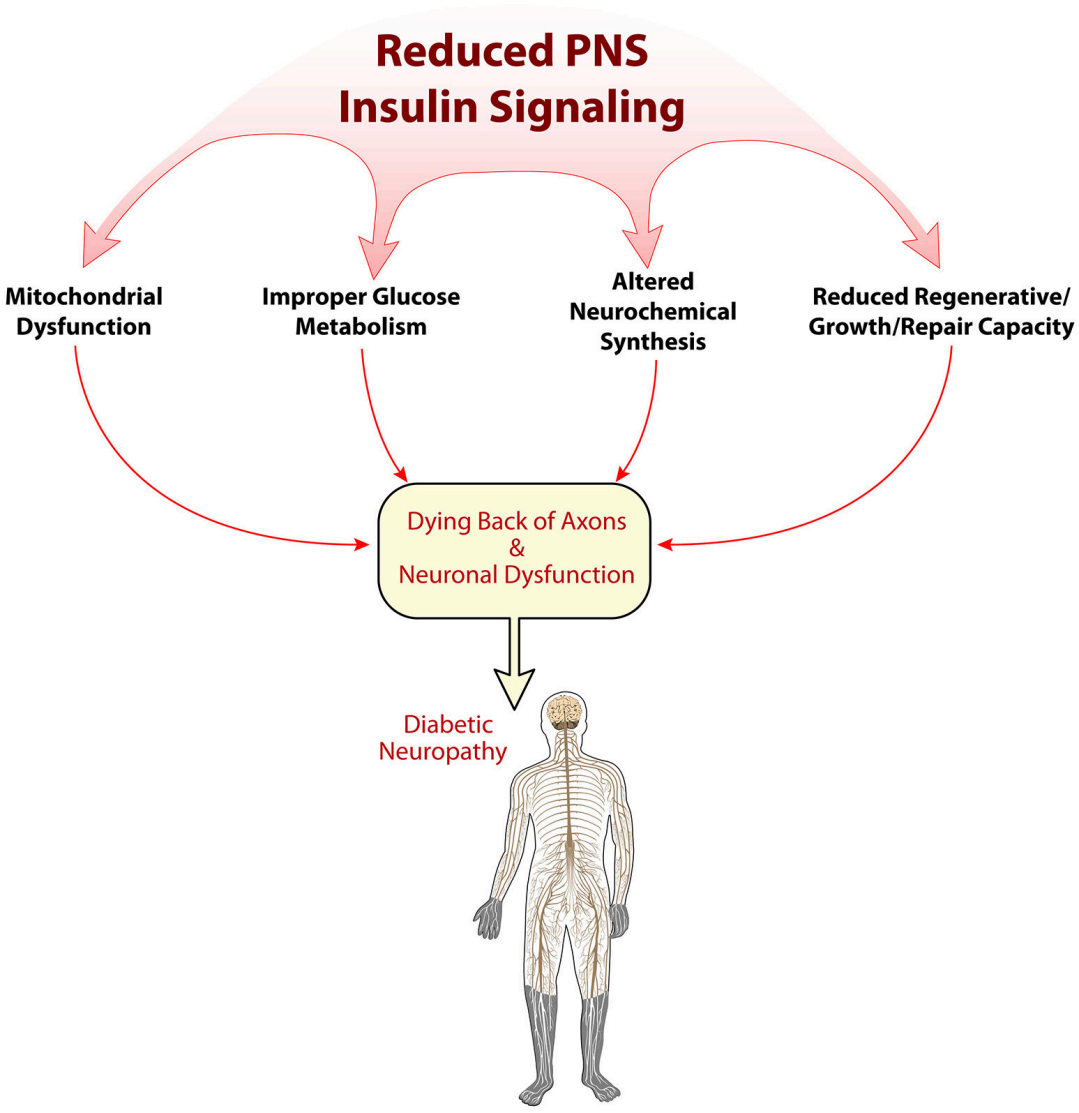
**Possible mechanisms of reduced insulin signaling in the pathogenesis of diabetic neuropathy**. Insulin's role in the peripheral nervous system is just beginning to be elucidated. Several early mechanisms of how alterations in insulin signaling may impact PNS physiology are shown here, including disruptions in mitochondrial function, metabolism, neurochemical synthesis, and regeneration/repair.

While the pathogenesis of DN is clearly related to hyperglycemia, there does appear to be non-glycemic triggers that also contribute to its etiology. The loss of normal neuronal insulin signaling in diabetes may be one of the main factors beyond hyperglycemia that plays a role in PNS dysfunction and neuropathic symptoms. It will also be important to consider that DN is a multifactorial disease, such that, perhaps hyperglycemic injury and reduced neuronal insulin signaling are not independent, but connected. Whereas, hyperglycemic injury causes neuronal damage, and that damage cannot be repaired due to reduced insulin support. The loss of appropriate PNS insulin support may result in impaired glucose metabolism, altered neuropeptide/neurotransmitter regulation, improper mitochondrial function, or reduced neurotrophic qualities, such as nerve regeneration (Figure 2). While the body of literature documenting decreased neuronal insulin signaling is expanding, it does appear that to definitively tease apart the effect that reduced insulin support has on neuronal function more powerful models are needed, such as conditional knockouts targeting insulin signaling in sensory neurons. In conclusion, although still a relatively new concept, dysfunctional neuronal insulin signaling may be a crucial component in the development of DN, and should be considered when investigating DN pathogenesis. Further research into this field could potentially highlight new therapeutic avenues and perhaps begin to provide relief for patients suffering from this devastating condition.

## Author contributions

CG: Primary author, performed literature review which was the basis of the submitted review. DW: Corresponding author, primary editor of the review as well as advisor of all work performed by CG.

## Funding

This work was supported by National Institutes of Health (NIH) grants R01NS43313 (DEW), P20 GM103418 from the Idea Network of Biomedical Research Excellence (INBRE) Program, and the Kansas IDDRC, U54 HD 090216.

### Conflict of interest statement

The authors declare that the research was conducted in the absence of any commercial or financial relationships that could be construed as a potential conflict of interest.

## References

[B3] Akter K. Lanza E. A. Martin S. A. Myronyuk N. Rua M. Raffa R. B. (2011). Diabetes mellitus and Alzheimer's disease: shared pathology and treatment? Br. J. Clin. Pharmacol. 71, 365–376. 10.1111/j.1365-2125.2010.03830.x 21284695 PMC3045545

[B4] Akude E. Zherebitskaya E. Chowdhury S. K. Smith D. R. Dobrowsky R. T. Fernyhough P. (2011). Diminished superoxide generation is associated with respiratory chain dysfunction and changes in the mitochondrial proteome of sensory neurons from diabetic rats. Diabetes 60, 288–297. 10.2337/db10-0818 20876714 PMC3012184

[B5] Apfel S. C. Schwartz S. Adornato B. T. Freeman R. Biton V. Rendell M.. (2000). Efficacy and safety of recombinant human nerve growth factor in patients with diabetic polyneuropathy: a randomized controlled trial. rhNGF Clinical Investigator Group. JAMA 284, 2215–2221. 10.1001/jama.284.17.2215 11056593

[B6] Baiou D. Santha P. Avelino A. Charrua A. Bacskai T. Matesz K.. (2007). Neurochemical characterization of insulin receptor-expressing primary sensory neurons in wild-type and vanilloid type 1 transient receptor potential receptor knockout mice. J. Comp. Neurol. 503, 334–347. 10.1002/cne.21389 17492627

[B7] Baura G. D. Foster D. M. Porte D. Jr. Kahn S. E. Bergman R. N. Cobelli C.. (1993). Saturable transport of insulin from plasma into the central nervous system of dogs *in vivo*. A mechanism for regulated insulin delivery to the brain. J. Clin. Invest. 92, 1824–1830. 10.1172/JCI116773 8408635 PMC288346

[B8] Bothwell M. (1982). Insulin and somatemedin MSA promote nerve growth factor-independent neurite formation by cultured chick dorsal root ganglionic sensory neurons. J. Neurosci. Res. 8, 225–231. 10.1002/jnr.490080212 6759678

[B9] Brussee V. Cunningham F. A. Zochodne D. W. (2004). Direct insulin signaling of neurons reverses diabetic neuropathy. Diabetes 53, 1824–1830. 10.2337/diabetes.53.7.1824 15220207

[B10] CDC National Diabetes Fact Sheet. (2014). National estimates and general information on diabetes and prediabetes in the united states (2011), in Centers for Disease Control and Prevention, 2011, ed C. US Department of Health and Human Services (Atlanta, GA: CDC, National Diabetes Fact Sheet), 1–12.

[B11] Chalk C. Benstead T. J. Moore F. (2007). Aldose reductase inhibitors for the treatment of diabetic polyneuropathy. Cochrane Database Syst. Rev. 2007:CD004572 10.1002/14651858.cd004572.pub2 PMC8406996 17943821

[B12] Chen D. K. Frizzi K. E. Guernsey L. S. Ladt K. Mizisin A. P. Calcutt N. A. (2013). Repeated monitoring of corneal nerves by confocal microscopy as an index of peripheral neuropathy in type-1 diabetic rodents and the effects of topical insulin. J. Peripher. Nerv. Syst. 18, 306–315. 10.1111/jns5.12044 24147903 PMC3981941

[B13] Cheng Z. Tseng Y. White M. F. (2010). Insulin signaling meets mitochondria in metabolism. Trends Endocrinol. Metab. 21, 589–598. 10.1016/j.tem.2010.06.005 20638297 PMC3994704

[B14] Chiu S. L. Chen C. M. Cline H. T. (2008). Insulin receptor signaling regulates synapse number, dendritic plasticity, and circuit function *in vivo*. Neuron 58, 708–719. 10.1016/j.neuron.2008.04.014 18549783 PMC3057650

[B15] Chiu S. L. Cline H. T. (2010). Insulin receptor signaling in the development of neuronal structure and function. Neural Dev. 5:7. 10.1186/1749-8104-5-7 20230616 PMC2843688

[B16] Choeiri C. Staines W. Messier C. (2002). Immunohistochemical localization and quantification of glucose transporters in the mouse brain. Neuroscience 111, 19–34. 10.1016/S0306-4522(01)00619-4 11955709

[B17] Chowdhury S. K. Zherebitskaya E. Smith D. R. Akude E. Chattopadhyay S. Jolivalt C. G.. (2010). Mitochondrial respiratory chain dysfunction in dorsal root ganglia of streptozotocin-induced diabetic rats and its correction by insulin treatment. Diabetes 59, 1082–1091. 10.2337/db09-1299 20103706 PMC2844817

[B18] Craft S. Baker L. D. Montine T. J. Minoshima S. Watson G. S. Claxton A.. (2012). Intranasal insulin therapy for Alzheimer disease and amnestic mild cognitive impairment: a pilot clinical trial. Arch. Neurol. 69, 29–38. 10.1001/archneurol.2011.233 21911655 PMC3260944

[B19] Datta S. R. Dudek H. Tao X. Masters S. Fu H. Gotoh Y.. (1997). Akt phosphorylation of BAD couples survival signals to the cell-intrinsic death machinery. Cell 91, 231–241. 10.1016/s0092-8674(00)80405-5 9346240

[B2] Diabetes Control Complications Trial Research Group (1995a). The effect of intensive diabetes therapy on the development, and progression of neuropathy. Ann. Intern. Med. 122, 561–568. 10.7326/0003-4819-122-8-199504150-00001 7887548

[B1] Diabetes Control Complications Trial Research Group (1995b). Effect of intensive diabetes treatment on nerve conduction in the diabetes control and complications trial. Ann. Neurol. 38, 869–880. 10.1002/ana.410380607 8526459

[B20] Dugan L. L. You Y. H. Ali S. S. Diamond-Stanic M. Miyamoto S. DeCleves A. E.. (2013). AMPK dysregulation promotes diabetes-related reduction of superoxide and mitochondrial function. J. Clin. Invest. 123, 4888–4899. 10.1172/jci66218 24135141 PMC3809777

[B21] Eckersley L. (2002). Role of the Schwann cell in diabetic neuropathy. Int. Rev. Neurobiol. 50, 293–321. 10.1016/S0074-7742(02)50081-7 12198814

[B22] Fadel J. R. Jolivalt C. G. Reagan L. P. (2013). Food for thought: the role of appetitive peptides in age-related cognitive decline. Ageing Res. Rev. 12, 764–776. 10.1016/j.arr.2013.01.009 23416469 PMC3774057

[B23] Fernyhough P. Mill J. F. Roberts J. L. Ishii D. N. (1989). Stabilization of tubulin mRNAs by insulin and insulin-like growth factor I during neurite formation. Brain Res. Mol. Brain Res. 6, 109–120. 10.1016/0169-328X(89)90044-2 2693875

[B24] Fernyhough P. Willars G. B. Lindsay R. M. Tomlinson D. R. (1993). Insulin and insulin-like growth factor I enhance regeneration in cultured adult rat sensory neurones. Brain Res. 607, 117–124. 10.1016/0006-8993(93)91496-F 8481790

[B25] Fex Svenningsen A. Kanje M. (1996). Insulin and the insulin-like growth factors I and II are mitogenic to cultured rat sciatic nerve segments and stimulate [3H]thymidine incorporation through their respective receptors. Glia 18, 68–72. 10.1002/(SICI)1098-1136(199609)18:1<68::AID-GLIA7>3.0.CO;2-# 8891693

[B26] Francis G. J. Martinez J. A. Liu W. Q. Zochodne D. W. Hanson L. R. Frey W. H. II. (2011). Motor end plate innervation loss in diabetes and the role of insulin. J. Neuropathol. Exp. Neurol. 70, 323–339. 10.1097/NEN.0b013e318215669a 21487310

[B27] Gerozissis K. (2008). Brain insulin, energy and glucose homeostasis; genes, environment and metabolic pathologies. Eur. J. Pharmacol. 585, 38–49. 10.1016/j.ejphar.2008.01.050 18407262

[B28] Greene D. A. Winegrad A. I. (1979). *In vitro* studies of the substrates for energy production and the effects of insulin on glucose utilization in the neural components of peripheral nerve. Diabetes 28, 878–887. 10.2337/diab.28.10.878 478182

[B29] Grillo C. A. Piroli G. G. Lawrence R. C. Wrighten S. A. Green A. J. Wilson S. P.. (2015). Hippocampal insulin resistance impairs spatial learning and synaptic plasticity. Diabetes 64, 3927–3936. 10.2337/db15-0596 26216852 PMC4613975

[B30] Grote C. W. Groover A. L. Ryals J. M. Geiger P. C. Feldman E. L. Wright D. E. (2013b). Peripheral nervous system insulin resistance in ob/ob mice. Acta Neuropathol. Commun. 1:15. 10.1186/2051-5960-1-15 24252636 PMC3893412

[B31] Grote C. W. Morris J. K. Ryals J. M. Geiger P. C. Wright D. E. (2011). Insulin receptor substrate 2 expression and involvement in neuronal insulin resistance in diabetic neuropathy. Exp. Diabetes Res. 2011:212571. 10.1155/2011/212571 21754917 PMC3132877

[B32] Grote C. W. Ryals J. M. Wright D. E. (2013a). *In vivo* peripheral nervous system insulin signaling. J, Periph. Nerv. Syst. 18, 209–219. 10.1111/jns5.12033 24028189 PMC4091841

[B33] Guo G. Kan M. Martinez J. A. Zochodne D. W. (2011). Local insulin and the rapid regrowth of diabetic epidermal axons. Neurobiol. Dis. 43, 414–421. 10.1016/j.nbd.2011.04.012 21530660

[B34] Habib A. A. Brannagan T. H. III. (2010). Therapeutic strategies for diabetic neuropathy. Curr. Neurol. Neurosci. Rep. 10, 92–100. 10.1007/s11910-010-0093-7 20425233

[B35] Haj-ali V. Mohaddes G. Babri S. H. (2009). Intracerebroventricular insulin improves spatial learning and memory in male Wistar rats. Behav. Neurosci. 123, 1309–1314. 10.1037/a0017722 20001114

[B36] Homs J. Pagès G. Ariza L. Casas C. Chillón M. Navarro X.. (2014). Intrathecal administration of IGF-I by AAVrh10 improves sensory and motor deficits in a mouse model of diabetic neuropathy. Mol. Ther. Methods Clin. Dev. 1:7. 10.1038/mtm.2013.7 26015946 PMC4365866

[B37] Hong S. Wiley J. W. (2005). Early painful diabetic neuropathy is associated with differential changes in the expression and function of vanilloid receptor 1. J. Biol. Chem. 280, 618–627. 10.1074/jbc.M408500200 15513920

[B38] Hoybergs Y. M. Meert T. F. (2007). The effect of low-dose insulin on mechanical sensitivity and allodynia in type I diabetes neuropathy. Neurosci. Lett. 417, 149–154. 10.1016/j.neulet.2007.02.087 17412508

[B39] Hu G. Jousilahti P. Bidel S. Antikainen R. Tuomilehto J. (2007). Type 2 diabetes and the risk of Parkinson's disease. Diabetes Care 30, 842–847. 10.2337/dc06-2011 17251276

[B40] Huang T. J. Price S. A. Chilton L. Calcutt N. A. Tomlinson D. R. Verkhratsky A.. (2003). Insulin prevents depolarization of the mitochondrial inner membrane in sensory neurons of type 1 diabetic rats in the presence of sustained hyperglycemia. Diabetes 52, 2129–2136. 10.2337/diabetes.52.8.2129 12882932

[B41] Huang T. J. Verkhratsky A. Fernyhough P. (2005). Insulin enhances mitochondrial inner membrane potential and increases ATP levels through phosphoinositide 3-kinase in adult sensory neurons. Mol. Cell. Neurosci. 28, 42–54. 10.1016/j.mcn.2004.08.009 15607940

[B42] Jolivalt C. G. Calcutt N. A. Masliah E. (2012). Similar pattern of peripheral neuropathy in mouse models of type 1 diabetes and Alzheimer's disease. Neuroscience 202, 405–412. 10.1016/j.neuroscience.2011.11.032 22178988 PMC3268846

[B43] Jolivalt C. G. Hurford R. Lee C. A. Dumaop W. Rockenstein E. Masliah E. (2010). Type 1 diabetes exaggerates features of Alzheimer's disease in APP transgenic mice. Exp. Neurol. 223, 422–431. 10.1016/j.expneurol.2009.11.005 19931251 PMC2864332

[B44] Jolivalt C. G. Lee C. A. Beiswenger K. K. Smith J. L. Orlov M. Torrance M. A.. (2008). Defective insulin signaling pathway and increased glycogen synthase kinase-3 activity in the brain of diabetic mice: parallels with Alzheimer's disease and correction by insulin. J. Neurosci. Res. 86, 3265–3274. 10.1002/jnr.21787 18627032 PMC4937800

[B45] Jones D. M. Tucker B. A. Rahimtula M. Mearow K. M. (2003). The synergistic effects of NGF and IGF-1 on neurite growth in adult sensory neurons: convergence on the PI 3-kinase signaling pathway. J. Neurochem. 86, 1116–1128. 10.1046/j.1471-4159.2003.01925.x 12911620

[B46] Kim B. McLean L. L. Philip S. S. Feldman E. L. (2011). Hyperinsulinemia induces insulin resistance in dorsal root ganglion neurons. Endocrinology 152, 3638–3647. 10.1210/en.2011-0029 21810948 PMC3176655

[B47] Kim S. J. Han Y. (2005). Insulin inhibits AMPA-induced neuronal damage via stimulation of protein kinase B (Akt). J. Neural. Transm. 112, 179–191. 10.1007/s00702-004-0163-6 15657639

[B48] King M. R. Anderson N. J. Liu C. Law E. Cundiff M. Mixcoatl-Zecuatl T. M.. (2015). Activation of the insulin-signaling pathway in sciatic nerve and hippocampus of type 1 diabetic rats. Neuroscience 303, 220–228. 10.1016/j.neuroscience.2015.06.060 26149351 PMC4532558

[B49] Leloup C. Arluison M. Kassis N. Lepetit N. Cartier N. Ferré P.. (1996). Discrete brain areas express the insulin-responsive glucose transporter GLUT4. Brain Res. Mol. Brain Res. 38, 45–53. 10.1016/0169-328X(95)00306-D 8737666

[B50] Le Roith D. Zick Y. (2001). Recent advances in our understanding of insulin action and insulin resistance. Diabetes Care 24, 588–597. 10.2337/diacare.24.3.588 11289486

[B51] Li Z. G. Zhang W. Sima A. A. (2003). C-peptide enhances insulin-mediated cell growth and protection against high glucose-induced apoptosis in SH-SY5Y cells. Diabetes Metab. Res. Rev. 19, 375–385. 10.1002/dmrr.389 12951645

[B52] Liang G. Cline G. W. Macica C. M. (2007). IGF-1 stimulates *de novo* fatty acid biosynthesis by Schwann cells during myelination. Glia 55, 632–641. 10.1002/glia.20496 17299765

[B53] Lilja J. Laulund F. Forsby A. (2007). Insulin and insulin-like growth factor type-I up-regulate the vanilloid receptor-1 (TRPV1) in stably TRPV1-expressing SH-SY5Y neuroblastoma cells. J. Neurosci. Res. 85, 1413–1419. 10.1002/jnr.21255 17385724

[B54] Luchsinger J. A. Tang M. X. Stern Y. Shea S. Mayeux R. (2001). Diabetes mellitus and risk of Alzheimer's disease and dementia with stroke in a multiethnic cohort. Am. J. Epidemiol. 154, 635–641. 10.1093/aje/154.7.635 11581097

[B55] McNay E. C. Ong C. T. McCrimmon R. J. Cresswell J. Bogan J. S. Sherwin R. S. (2010). Hippocampal memory processes are modulated by insulin and high-fat-induced insulin resistance. Neurobiol. Learn. Mem. 93, 546–553. 10.1016/j.nlm.2010.02.002 20176121 PMC2878207

[B56] Murakawa Y. Zhang W. Pierson C. R. Brismar T. Ostenson C. G. Efendic S.. (2002). Impaired glucose tolerance and insulinopenia in the GK-rat causes peripheral neuropathy. Diabetes Metab. Res. Rev. 18, 473–483. 10.1002/dmrr.326 12469361

[B57] Obici S. Feng Z. Karkanias G. Baskin D. G. Rossetti L. (2002a). Decreasing hypothalamic insulin receptors causes hyperphagia and insulin resistance in rats. Nat. Neurosci. 5, 566–572. 10.1038/nn0602-861 12021765

[B58] Obici S. Zhang B. B. Karkanias G. Rossetti L. (2002b). Hypothalamic insulin signaling is required for inhibition of glucose production. Nat. Med. 8, 1376–1382. 10.1038/nm1202-798 12426561

[B59] Ogata T. Iijima S. Hoshikawa S. Miura T. Yamamoto S. Oda H.. (2004). Opposing extracellular signal-regulated kinase and Akt pathways control Schwann cell myelination. J. Neurosci. 24, 6724–6732. 10.1523/jneurosci.5520-03.2004 15282275 PMC6729716

[B60] Pabbidi R. M. Yu S. Q. Peng S. Khardori R. Pauza M. E. Premkumar L. S. (2008). Influence of TRPV1 on diabetes-induced alterations in thermal pain sensitivity. Mol. Pain 4:9. 10.1186/1744-8069-4-9 18312687 PMC2275252

[B61] Park C. R. Seeley R. J. Craft S. Woods S. C. (2000). Intracerebroventricular insulin enhances memory in a passive-avoidance task. Physiol. Behav. 68, 509–514. 10.1016/S0031-9384(99)00220-6 10713291

[B62] Patel N. J. Llewelyn J. G. Wright D. W. Thomas P. K. (1994). Glucose and leucine uptake by rat dorsal root ganglia is not insulin sensitive. J. Neurol. Sci. 121, 159–162. 10.1016/0022-510X(94)90345-X 8158208

[B63] Pedersen W. A. McMillan P. J. Kulstad J. J. Leverenz J. B. Craft S. Haynatzki G. R. (2006). Rosiglitazone attenuates learning and memory deficits in Tg2576 Alzheimer mice. Exp. Neurol. 199, 265–273. 10.1016/j.expneurol.2006.01.018 16515786

[B64] Plum L. Schubert M. Brüning J. C. (2005). The role of insulin receptor signaling in the brain. Trends Endocrinol. Metab. 16, 59–65. 10.1016/j.tem.2005.01.008 15734146

[B65] Rachana K. S. Manu M. S. Advirao G. M. (2016). Insulin influenced expression of myelin proteins in diabetic peripheral neuropathy. Neurosci. Lett. 629, 110–115. 10.1016/j.neulet.2016.06.067 27373589

[B66] Rauskolb S. Dombert B. Sendtner M. (2017). Insulin-like growth factor 1 in diabetic neuropathy and amyotrophic lateral sclerosis. Neurobiol. Dis. 97, 103–113. 10.1016/j.nbd.2016.04.007 27142684

[B67] Recio-Pinto E. Lang F. F. Ishii D. N. (1984). Insulin and insulin-like growth factor II permit nerve growth factor binding and the neurite formation response in cultured human neuroblastoma cells. Proc. Natl. Acad. Sci. U.S.A. 81, 2562–2566. 10.1073/pnas.81.8.2562 6326132 PMC345103

[B68] Recio-Pinto E. Rechler M. M. Ishii D. N. (1986). Effects of insulin, insulin-like growth factor-II, and nerve growth factor on neurite formation and survival in cultured sympathetic and sensory neurons. J. Neurosci. 6, 1211–1219. 3519887 10.1523/JNEUROSCI.06-05-01211.1986 PMC6568556

[B69] Ribe E. M. Lovestone S. (2016). Insulin signalling in Alzheimer's disease and diabetes: from epidemiology to molecular links. J. Intern. Med. 280, 430–442. 10.1111/joim.12534 27739227

[B70] Romanovsky D. Cruz N. F. Dienel G. A. Dobretsov M. (2006). Mechanical hyperalgesia correlates with insulin deficiency in normoglycemic streptozotocin-treated rats. Neurobiol. Dis. 24, 384–394. 10.1016/j.nbd.2006.07.009 16935517

[B71] Romanovsky D. Wang J. Al-Chaer E. D. Stimers J. R. Dobretsov M. (2010). Comparison of metabolic and neuropathy profiles of rats with streptozotocin-induced overt and moderate insulinopenia. Neuroscience 170, 337–347. 10.1016/j.neuroscience.2010.06.059 20600635 PMC2926213

[B72] Sathianathan V. Avelino A. Charrua A. Santha P. Matesz K. Cruz F.. (2003). Insulin induces cobalt uptake in a subpopulation of rat cultured primary sensory neurons. Eur. J. Neurosci. 18, 2477–2486. 10.1046/j.1460-9568.2003.03004.x 14622148

[B73] Sato T. Hanyu H. Hirao K. Kanetaka H. Sakurai H. Iwamoto T. (2011). Efficacy of PPAR-gamma agonist pioglitazone in mild Alzheimer disease. Neurobiol. Aging 32, 1626–1633. 10.1016/j.neurobiolaging.2009.10.009 19923038

[B74] Schubert M. Gautam D. Surjo D. Ueki K. Baudler S. Schubert D.. (2004). Role for neuronal insulin resistance in neurodegenerative diseases. Proc. Natl. Acad. Sci. U.S.A. 101, 3100–3105. 10.1073/pnas.0308724101 14981233 PMC365750

[B75] Shettar A. Muttagi G. (2012). Developmental regulation of insulin receptor gene in sciatic nerves and role of insulin on glycoprotein P0 in the Schwann cells. Peptides 36, 46–53. 10.1016/j.peptides.2012.04.012 22564491

[B76] Shetter A. R. Muttagi G. Sagar C. B. (2011). Expression and localization of insulin receptors in dissociated primary cultures of rat Schwann cells. Cell Biol. Int. 35, 299–304. 10.1042/CBI20100523 20977434

[B77] Simon C. M. Rauskolb S. Gunnersen J. M. Holtmann B. Drepper C. Dombert B.. (2015). Dysregulated IGFBP5 expression causes axon degeneration and motoneuron loss in diabetic neuropathy. Acta Neuropathol. 130, 373–387. 10.1007/s00401-015-1446-8 26025657 PMC4541707

[B78] Singh B. Xu Y. McLaughlin T. Singh V. Martinez J. A. Krishnan A.. (2012). Resistance to trophic neurite outgrowth of sensory neurons exposed to insulin. J. Neurochem. 121, 263–276. 10.1111/j.1471-4159.2012.07681.x 22303986

[B79] Skaper S. D. Selak I. Varon S. (1982). Molecular requirements for survival of cultured avian and rodent dorsal root ganglionic neurons responding to different trophic factors. J. Neurosci. Res. 8, 251–261. 10.1002/jnr.490080215 7154114

[B80] Snyder E. Y. Kim S. U. (1980). Insulin: is it a nerve survival factor? Brain Res. 196, 565–571. 10.1016/0006-8993(80)90426-6 6994854

[B81] Sokoloff L. (1973). Metabolism of ketone bodies by the brain. Annu. Rev. Med. 24, 271–280. 10.1146/annurev.me.24.020173.001415 4575857

[B82] Song Z. Fu D. T. Chan Y. S. Leung S. Chung S. S. Chung S. K. (2003). Transgenic mice overexpressing aldose reductase in Schwann cells show more severe nerve conduction velocity deficit and oxidative stress under hyperglycemic stress. Mol. Cell. Neurosci. 23, 638–647. 10.1016/S1044-7431(03)00096-4 12932443

[B83] Spanswick D. Smith M. A. Mirshamsi S. Routh V. H. Ashford M. L. (2000). Insulin activates ATP-sensitive K+ channels in hypothalamic neurons of lean, but not obese rats. Nat. Neurosci. 3, 757–758. 10.1038/77660 10903566

[B84] Spicarova D. Palecek J. (2010). Modulation of AMPA excitatory postsynaptic currents in the spinal cord dorsal horn neurons by insulin. Neuroscience 166, 305–311. 10.1016/j.neuroscience.2009.12.007 20005924

[B85] Sugimoto K. Baba M. Suzuki S. Yagihashi S. (2013). The impact of low-dose insulin on peripheral nerve insulin receptor signaling in streptozotocin-induced diabetic rats. PLoS ONE 8:e74247. 10.1371/journal.pone.0074247 24023699 PMC3758356

[B86] Sugimoto K. Murakawa Y. Sima A. A. (2002). Expression and localization of insulin receptor in rat dorsal root ganglion and spinal cord. J. Periph. Nerv. Syst. 7, 44–53. 10.1046/j.1529-8027.2002.02005.x 11939351

[B87] Sugimoto K. Murakawa Y. Zhang W. Xu G. Sima A. A. (2000). Insulin receptor in rat peripheral nerve: its localization and alternatively spliced isoforms. Diabetes Metab. Res. Rev. 16, 354–363. 10.1002/1520-7560(200009/10)16:5<354::AID-DMRR149>3.0.CO;2-H 11025559

[B88] Sugimoto K. Rashid I. B. Shoji M. Suda T. Yasujima M. (2008). Early changes in insulin receptor signaling and pain sensation in streptozotocin-induced diabetic neuropathy in rats. J. Pain 9, 237–245. 10.1016/j.jpain.2007.10.016 18331706

[B89] Takamoto I. Terauchi Y. Kubota N. Ohsugi M. Ueki K. Kadowaki T. (2008). Crucial role of insulin receptor substrate-2 in compensatory beta-cell hyperplasia in response to high fat diet-induced insulin resistance. Diabetes Obes Metab 10(Suppl. 4), 147–156. 10.1111/j.1463-1326.2008.00951.x 18834442

[B90] Talbot K. Wang H. Y. Kazi H. Han L. Y. Bakshi K. P. Stucky A.. (2012). Demonstrated brain insulin resistance in Alzheimer's disease patients is associated with IGF-1 resistance, IRS-1 dysregulation, and cognitive decline. J. Clin. Invest. 122, 1316–1338. 10.1172/JCI59903 22476197 PMC3314463

[B91] Taniguchi C. M. Emanuelli B. Kahn C. R. (2006). Critical nodes in signalling pathways: insights into insulin action. Nat.Rev. Mol. Cell Biol. 7, 85–96. 10.1038/nrm1837 16493415

[B92] Tomlinson D. R. Gardiner N. J. (2008). Glucose neurotoxicity. Nat. Rev. Neurosci. 9, 36–45. 10.1038/nrn2294 18094705

[B93] Toth C. Brussee V. Martinez J. A. McDonald D. Cunningham F. A. Zochodne D. W. (2006a). Rescue and regeneration of injured peripheral nerve axons by intrathecal insulin. Neuroscience 139, 429–449. 10.1016/j.neuroscience.2005.11.065 16529870

[B94] Toth C. Brussee V. Zochodne D. W. (2006b). Remote neurotrophic support of epidermal nerve fibres in experimental diabetes. Diabetologia 49, 1081–1088. 10.1007/s00125-006-0169-8 16528572

[B95] Van Buren J. J. Bhat S. Rotello R. Pauza M. E. Premkumar L. S. (2005). Sensitization and translocation of TRPV1 by insulin and IGF-I. Mol. Pain 1:17. 10.1186/1744-8069-1-17 15857517 PMC1142339

[B96] Vincent A. M. Callaghan B. C. Smith A. L. Feldman E. L. (2011). Diabetic neuropathy: cellular mechanisms as therapeutic targets. Nat. Rev. Neurol. 7, 573–583. 10.1038/nrneurol.2011.137 21912405

[B97] Wang Y. Y. Lui P. C. Li J. Y. (2009). Receptor-mediated therapeutic transport across the blood-brain barrier. Immunotherapy 1, 983–993. 10.2217/imt.09.75 20635914

[B98] White M. F. (2002). IRS proteins and the common path to diabetes. Am. J. Physiol. Endocrinol. Metab. 283, E413–E422. 10.1152/ajpendo.00514.2001 12169433

[B99] White M. F. (2006). Regulating insulin signaling and beta-cell function through IRS proteins. Can. J. Physiol. Pharmacol. 84, 725–737. 10.1139/y06-008 16998536

[B100] Wild S. Roglic G. Green A. Sicree R. King H. (2004). Global prevalence of diabetes: estimates for the year 2000 projections for 2030. Diabetes Care 27, 1047–1053. 10.2337/diacare.27.5.1047 15111519

[B101] Xu Q. G. Li X. Q. Kotecha S. A. Cheng C. Sun H. S. Zochodne D. W. (2004). Insulin as an *in vivo* growth factor. Exp. Neurol. 188, 43–51. 10.1016/j.expneurol.2004.03.008 15191801

[B102] Zhang L. Yu C. Vasquez F. E. Galeva N. Onyango I. Swerdlow R. H.. (2010). Hyperglycemia alters the schwann cell mitochondrial proteome and decreases coupled respiration in the absence of superoxide production. J. Proteome Res. 9, 458–471. 10.1021/pr900818g 19905032 PMC2801777

[B103] Zochodne D. W. (2007). Diabetes mellitus and the peripheral nervous system: manifestations and mechanisms. Muscle Nerve 36, 144–166. 10.1002/mus.20785 17469109

[B104] Zochodne D. W. (2014). Mechanisms of diabetic neuron damage: molecular pathways. Handb. Clin. Neurol. 126, 379–399. 10.1016/B978-0-444-53480-4.00028-X 25410235

[B105] Zochodne D. W. (2015). Diabetes and the plasticity of sensory neurons. Neurosci. Lett. 596, 60–65. 10.1016/j.neulet.2014.11.017 25445357

